# TALE and Shape: How to Make a Leaf Different

**DOI:** 10.3390/plants2020317

**Published:** 2013-05-06

**Authors:** Elisabetta Di Giacomo, Maria Adelaide Iannelli, Giovanna Frugis

**Affiliations:** Istituto di Biologia e Biotecnologia Agraria, UOS Roma, Consiglio Nazionale delle Ricerche, Via Salaria Km. 29,300, Monterotondo Scalo, 00015 Roma, Italy; E-Mails: elisabetta.digiacomo@mlib.ibba.cnr.it (E.D.G.); iannelli@ibba.cnr.it (M.A.I.)

**Keywords:** TALE, BLH, KNOX, homeobox transcription factors, target genes, leaf development, vascular development, cell fate, cell differentiation, shoot apical meristem

## Abstract

The Three Amino acid Loop Extension (TALE) proteins constitute an ancestral superclass of homeodomain transcription factors conserved in animals, plants and fungi. In plants they comprise two classes, KNOTTED1-LIKE homeobox (KNOX) and BEL1-like homeobox (BLH or BELL, hereafter referred to as BLH), which are involved in shoot apical meristem (SAM) function, as well as in the determination and morphological development of leaves, stems and inflorescences. Selective protein-protein interactions between KNOXs and BLHs affect heterodimer subcellular localization and target affinity. KNOXs exert their roles by maintaining a proper balance between undifferentiated and differentiated cell state through the modulation of multiple hormonal pathways. A pivotal function of KNOX in evolutionary diversification of leaf morphology has been assessed. In the SAM of both simple- and compound-leafed seed species, downregulation of most class 1 *KNOX* (*KNOX1*) genes marks the sites of leaf primordia initiation. However, *KNOX1* expression is re-established during leaf primordia development of compound-leafed species to maintain transient indeterminacy and morphogenetic activity at the leaf margins. Despite the increasing knowledge available about KNOX1 protein function in plant development, a comprehensive view on their downstream effectors remains elusive. This review highlights the role of TALE proteins in leaf initiation and morphological plasticity with a focus on recent advances in the identification of downstream target genes and pathways.

## 1. Introduction

Homeodomain (HD) transcription factors play major roles in the development of complex organisms, ranging from humans to plants. Increasing morphological complexity requires finely tuned processes of polarized growth along different axis to establish specialized organs with determinate shapes and symmetries. This process is fundamental during development and involves the integration of multiple signals from different parts of the organism’s body. 

Several classes of HD proteins participate in developmental signal integration and coordinated growth in complex organisms, among these the TALE (Three Amino acid Loop Extension) superclass is characterized by three extra amino acids between helix 1 and helix 2 of the homeodomain [1,2,3]. In animals, among the four subgroups of TALEs identified, the members of MEIS/PREP and Pre-B cell leukaemia transcription factors (PBX) form heterotrimers with homeobox proteins (Hox). Together they act within transcriptional regulation complexes to specify anteroposterior identities and regulate genetic programs during development [4]. Besides the role of PBX/MEIS interactions in heterodimer nuclear targeting and in increasing DNA binding specificity of PBX/HOX/MEIS complexes [5,6], a Hox-independent role of PBXs has been recently proposed in integrating transduction signals to regulate gene expression programs [4]. In higher plants, TALE superclass comprises two subgroups of proteins, the KNOTTED1-LIKE homeobox (KNOX) and BEL1-like homeobox (BLH or BELL, hereafter referred to as BLH) [3]. Similar to animal TALEs, BLH/KNOX heterodimerization is functional to nuclear localization [7,8] and to DNA binding affinity [9]. However, no specific interaction with other non-TALE homeodomain proteins has been described so far. Interaction with MADS homeotic, but not homeodomain proteins, has been reported in flower development [10]. 

**Figure 1:**
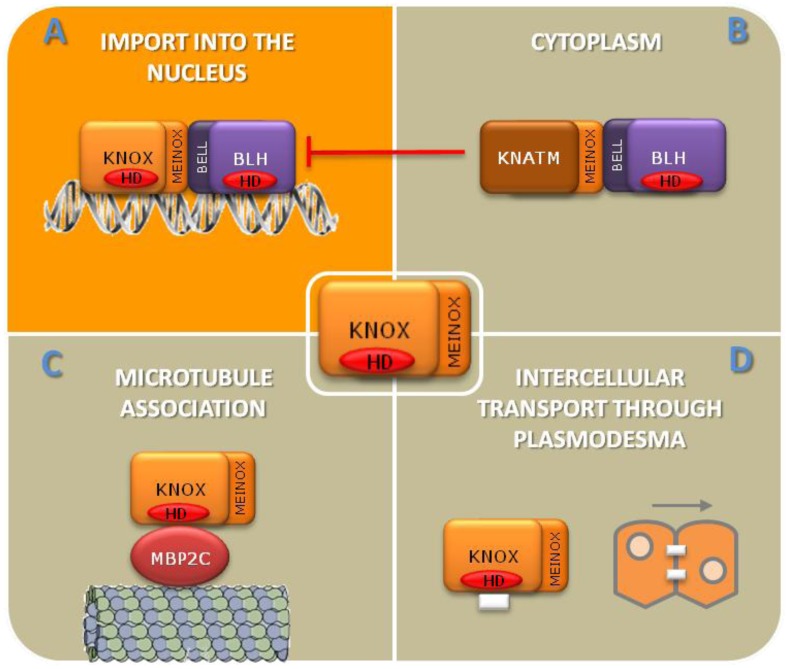
KNOTTED1-LIKE homeobox (KNOX) intracellular localization is regulated by protein-protein interactions. KNOX/BLH interaction through the MEINOX and BELL domains, respectively, targets the complex to the nucleus (**A**). KNATM interacts with someBEL1-like homeobox (BLHs) through its MEINOX domain and interferes with KNOX/BLH complex formation and nuclear import (**B**). Interaction with the microtubule-associated protein MBP2C targets KNOX to the plasma membrane (**C**) [32]. Some KNOXs are able to move from cell to cell through plasmodesmata (**D**) [33,34,35,36].

TALEs are encoded by two small subfamilies in the *Arabidopsis thaliana* genome where the sequences of eight *KNOX* and 13 *BLH* genes have been identified [11]. *KNOX* genes are further subdivided into two phylogenetic classes. *STM*, *KNAT1/BP*, *KNAT2* and *KNAT6* fall into class 1 (hereafter referred to as *KNOX1*) while *KNAT3*, *KNAT4*, *KNAT5* and *KNAT7* into class 2 (hereafter referred to as *KNOX2*). These two classes are characterized by specific intron number and position, structural similarities in the consensus domain outside the homeobox and by different expression pathways [12,13]. Recently, a novel *KNOX* lacking the homeobox, *KNATM*, was identified in the Arabidopsis genome [14]. *KNOX1* transcripts are less abundant, with a more localized expression in specific domains of shoot apical meristems (SAMs), whereas *KNOX2* genes are expressed in most plant organs. *KNATM* is expressed in proximal-lateral domains of organ primordia, at the boundary of mature organs and in leaf hydathodes. As mentioned before, members of KNOX and BLH proteins can physically interact (Table 1) through a bi-partite consensus domain located upstream of the HD, namely MEINOX and BEL respectively, and this interaction is functional to KNOX/BLH heterodimer translocation from cytoplasm into the nucleus (Figure 1) [7,8]. A matter of debate is whether KNOX and BLH can exert some of their functions independently of each other or whether the formation of KNOX/BLH heterodimers is mandatory for TALEs to work. 

**Table 1:** Interaction between *A. thaliana* KNOX and BLH proteins. KNOTTED1-LIKE homeobox (KNOX) proteins are indicated in rows and BEL1-like homeobox (BLH) proteins in columns with corresponding gene ID numbers. Interaction data deriving from both low and high throughput two-hybrid screens are indicated in grey, interactions that were confirmed *in planta* are highlighted in green.

		KNOX								
		STM	KNAT1/BP	KNAT2	KNAT6	KNAT3	KNAT4	KNAT5	KNAT7	KNATM
		AT1G62360	AT4G08150	AT1G70510	AT1G23380	AT5G25220	AT5G11060	AT4G32040	AT1G62990	AT1G14760
**BLH/BELL**	**ATH1**	[8]	[8]	[15]	[15]		[16]		[16]	
	**AT4G32980**									
	**BEL1**	[16,17,18]	[16,18]	[16,17]	[16]			[17]		[14]
	**AT5G41410**									
	**BLH1**	[16]			[16]	[16]		[16]		
	**AT2G35940**									
	**BLH2/**SAW1	[16]	[16]	[16]	[16]	[16]		[16]		[14]
	**AT4G36870**									
	**BLH3**	[8]	[8]		[16]	[16]		[16]		
	**AT1G75410**									
	**BLH4/**SAW2	[16]	[16]	[16]	[16]	[16]		[16]		[14]
	**AT2G23760**									
	**BLH5**			[16]	[16]	[16]		[16]	[16]	
	**AT2G27220**									
	**BLH6**	[16]	[16]	[16]	[16]	[16]	[16]	[16]		
	**AT4G34610**									
	**BLH7**		[16,19]		[16]	[16]		[16]	[16]	
	**AT2G16400**									
	**BLH8**/PNF	[18]	[16,18]	[16]						
	**AT2G27990**									
	**BLH9**/PNY/BLR	[8]	[8]	[16]	[7,16]	[16]		[16]		[14]
	**AT5G020309**									
	**BLH10**							[16]		
	**AT1G19700**									
	**BLH11**									
	**AT1G75430**									

Plant KNOX1 proteins have been largely characterized in monocotyledons and dicotyledons as key players in developmental processes where a fine equilibrium between undifferentiated and differentiated cell fate is required [20,21]. Most interestingly, KNOX1 play a pivotal role in evolutionary diversification of leaf morphology, as different expression patterns in leaves coincide with different degrees of leaf lamina serration and complexity [22]. Members of the BLH family may act redundantly as interacting partners of KNOX to regulate plant development [23]. However, very little is known about the functional meaning of KNOX/BLH interactions or the extent to which they contribute to specific developmental processes in plants. 

In this review, we summarize the current evidence for a critical role of TALEs in leaf development. For more information addressing the contribution of KNOX to the evolution of leaf morphology we refer the reader to several reviews recently published [24,25,26,27,28,29,30]. A detailed overview of TALE function in other important developmental processes lies outside the scope of the present review [21,23,31]. However, we will introduce the role of TALEs in the shoot apical meristem (SAM) to discuss molecular mechanisms shared by leaf shape determination and organogenesis at the SAM. We will critically discuss novel findings regarding the identification of direct or indirect target genes and pathways that may act downstream of TALEs in leaf formation and development.

## 2. Overview of the Role of TALEs in Shoot Apical Meristem

The first plant homeobox gene was identified in the laboratory of Sarah Hake [37] through the molecular characterization of *knotted1* (*kn1*), a gain-of-function mutant of maize that displayed transformation of leaf blade cellular fate towards sheath-like identity. The first evidence for a KNOX1 involvement in SAM formation and meristem boundaries maintenance derived from the characterization of a recessive mutation in a *KN1-like* Arabidopsis gene, *SHOOT MERISTEMLESS* (*STM*) [38,39]. Plants carrying the *stm-1* allele in *A. thaliana* Landsberg background were unable to form or maintain a functional SAM and only produced two cotyledons, often fused at their base. Plants harbouring weak alleles of *stm* or RNA interference (RNAi) constructs in *A. thaliana* Columbia background revealed an additional role of *STM* in vegetative-to-reproductive phase change as well as inflorescence and flower meristem formation [40,41,42,43]. These *stm* mutants can be fully phenocopied by the triple mutant that combined lesions in the *BLH* genes *ARABIDOPSIS THALIANA HOMEOBOX 1 (ATH1*), *PENNYWISE (PNY)* and *POUND-FOOLISH* (*PNF*). Hence, the observed meristem defects may result from loss of combinatorial BLH-STM heterodimers control [44]. Among the three *BLH* genes involved in SAM maintenance, *ATH1* mainly contributes to the vegetative meristem function whereas the *PNY* and *PNF* roles prevail in inflorescence stem architecture and flower development [44]. Studies on both maize *KN1* null and gain-of-function mutants, suggested that KN1 regulates leaf proximal identity and that when KN1 is absent leaves may fail to form because the proximal boundary is not defined [45]. Both *KN1* and *STM* are expressed in the central zone (CZ) and peripheral zone (PZ) of the SAM but are downregulated at the site of leaf primordia initiation (P0). Besides its involvement in SAM formation, STM may contribute to maintain a boundary between the CZ and PZ [31,40] and to regulate the allocation of cells into initiating organ primordia [46]. In the PZ, KN1/STM may act to define the meristem-organ boundary, which separates the meristem from the developing leaf [12,31,47,48,49,50]. Studies of other KNOX1 transcription factors in Arabidopsis have suggested that KNAT2, KNAT6 and KNAT1/BP proteins may contribute redundantly to meristem and meristem-organ boundaries maintenance [23]. 

## 3. TALE Contribution to Leaf Initiation and Morphology in Simple and Compound-Leafed Plant Species

**Figure 2:**
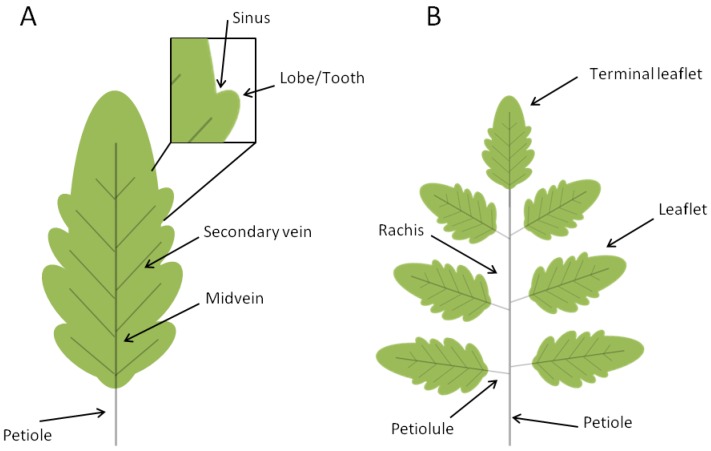
Schematic representation of simple and compound leaves. Leaves are traditionally divided into two major morphogenetic classes: simple (**A**) and compound (**B**). Simple leaves have a single flat blade/lamina, the margins of which are continuous and may be smooth, lobed, or serrated (with asymmetrical teeth pointing forward). The blades of compound leaves of eudicot plants are composed of several regularly spaced sessile or petiolated appendages called leaflets that are attached to a central rachis, the latter corresponding to the middle vein of a simple leaf. The leaf blade, or the rachis in compound leaves, is attached to the stem by the petiole.

The leaves of seed plants evolved from a primitive shoot system as determinate growth organs that arise at the flank of the shoot apical meristem. Leaves are formed by a structural vascular net (leaf veins), which is continuous with the shoot vascular bundles, and a lamina with dorsiventral, mediolateral and proximal-distal polarities. The blade of dicot leaves is intersected by an intricate arrangement of successive orders of branched veins with a central primary vein (midvein) that develops acropetally at the center of the leaf and extends along the whole leaf length. Differently, leaves from monocots are characterized by a parallel vein system. Leaves display an enormous degree of morphological variability and are mainly classified into simple and compound. Simple leaves consist of a single leaf blade while compound leaves bear several leaflets departing from a major axis, named rachis (Figure 2).

Leaf development encompasses three continuous and overlapping phases [26,51,52,53,54,55]. During the first phase of leaf initiation, the leaf primordium emerges from the flanks of the SAM at positions determined by specific phyllotactic patterns. In the second phase, the leaf expands laterally and primary morphogenesis (PM) events occur from specific meristematic regions at the leaf margin (blastozones) [56]. In the third phase of secondary morphogenesis (SM), extensive cell expansion and histogenesis occurs.

TALE transcription factors have been involved in key morphogenetic processes occurring at early stages of leaf development: (i) leaf primodia initiation; (ii) formation of leaf serrations; (iii) leaflet formation in compound leaves.

### 3.1. TALE Genes in Organ Primordia Initiation

Leaf primordia initiate at the SAM, in a species-dependent phyllotactic pattern, at specific sites marked by auxin maxima. Organ outgrowth is preceded by cell microtubule rearrangement and the entire sequence of events leading to leaf formation can be triggered by local changes in the cell expansion rate [57]. This was shown by experiments where local external application, or expression of the cell wall lubricating protein expansins, could induce organ outgrowth from the meristem [58,59,60]. Recently, an involvement of enzymes that modify the methyl-esterification of the pectin cell wall component in organ outgrowth has been shown [61,62], further implicating cell wall modification in the organogenesis processes at the SAM. In both simple and compound leaves, downregulation of *KNOX1* genes marks early the sites of leaf primordia initiation. It has been suggested that *KNOXs* downregulation may be functional to organ formation, acquisition of lateral organ identity and cell differentiation [20]. However, ectopic expression of *KNOX1* under the control of CaMV 35S promoter in plant tissues including leaf primordia, does not prevent leaf initiation but dramatically alters the vascular venation pattern and the morphology of leaves. Lobes or indeterminate structures form at the margin of simple-leafed species while increased lamina dissection is observed in compound leaves (see Subsection 3.2) [20]. Moreover, when *STM* was expressed in leaf primordia under the control of *AINTEGUMENTA* (*ANT*) promoter, which is active in primordia of cotyledons and leaves [63], the observed phenotype was similar to that of *35S::STM* plants. The leaves were lobed and smaller than in the wild type, and in the most extreme cases, were reduced to small finger-like structures [64]. Conversely, overexpression of *WUSCHEL* (*WUS*), another major regulator of SAM formation and maintenance, under the control of the *ANT* promoter, resulted in plants with an enlarged meristem that lack lateral organ formation [64]. These studies suggest that misexpression of *KNOX1* in leaf initials would not prevent organ outgrowth but would rather affect organ boundary formation, later stages of cell differentiation and establishment of leaf vein patterning. Nonetheless, a role for *KNOX1* downregulation in organ outgrowth cannot be excluded. Post-transcriptional events such as mRNA and protein instability or protein-protein interactions may impede KNOX1 activity in leaf founder cells even when ectopically expressed. Most importantly, expression of KNOX1 proteins in leaf initials might be ineffective due to lack of proper BLH interactors. In fact, the few studies on mRNA localization of *BLH* genes, such as *BELLRINGER (BLR/BLH9)*, showed *BLH* downregulation at the site of organ primordia initiation [65]. 

Very recently, a new *BLR/BLH9* mutant, *blr-6*, was found to bear ectopic flowers at the shoot apex and altered phyllotaxis, together with reduced pectin methylesterification in the meristem caused by the upregulation of the *PECTIN METHYLESTERASE 5* (*PME5)* gene [62]. Complete restoration of meristem phyllotaxis was observed in *blr-6/pme5-1* double mutant, thus showing that the altered phyllotaxis in the *blr-6* mutant in the meristem could be explained entirely by the ectopic *PME5* expression in the meristem dome. These studies point to pectin demethylesterification as a critical step in the loss of meristem identity and in the allocation of the cell to an organ primordium. The function of BLH9 would therefore be to inhibit *PME* in the shoot meristem, which is consistent with the observation that overexpression of a PME inhibitor (PMEI3) completely prevents primordia formation [61]. 

### 3.2. TALE Genes in the Control of Simple Leaf Morphology

Margins of simple leaves can be lobed, serrated, or smooth. Leaf margin morphogenesis is primarily achieved through the regulation of differential growth of the leaf blade at the peripheral zone of the SAM. A putative role for TALE proteins in leaf marginal outgrowth has been suggested by *KNOX* overexpression studies that showed formation of highly lobed leaves and marginal outgrowth in different simple-leafed plant species [20]. For instance, leaves of plants expressing *KNAT1/BP* from the 35S promoter are lobed and folded upwards with ectopic stipules and meristems in the sinus regions [66]. During a screen to identify genes that negatively regulated *KNOX* expression *asymmetric leaves1* (*as1*), *asymmetric leaves 2* (*as2*) and *serrate* (*se*) Arabidopsis mutants were isolated [67]. The *se* mutant displayed strong leaf serration whereas *as1* and *as2* showed rumpled rosette leaves reminiscent of tobacco leaves overexpressing *KNAT1* [67]. Indeed, double *as1/as2* mutants displayed misexpression of *KNAT1/BP* and *KNAT2* in the leaves whereas *se* increased *as1/as2* leaf lobing phenotype although no misexpression of *KNAT1/BP* in this single mutant was observed. A direct link between TALEs and leaf serration was further established through the characterization of two *BLH* genes, *SAWTOOTH1* (*SAW1*) and *SAW2*, formerly called *BLH2* and *BLH4*, respectively [68]. The *saw1-1 saw2-1* Arabidopsis double mutants showed increased serrations in leaf margins and revolute leaves with margins curled abaxially. This phenotype was accompanied by reactivation of the *KNOX* genes *KNAT1/BP* and *KNAT2* in the leaves [68]. SAW1 interacts with STM, KNAT1/BP, KNAT2, KNAT3, KNAT6 and KNAT5 and was shown to repress *KNAT1/BP* expression in leaf hydathodes [68], differently from *asymmetric* mutants where *KNOX* reactivation was observed in leaf petioles and vascular veins [67]. KNATM, the KNOX protein lacking the homeobox, interacts with the BLH proteins SAW1, SAW2, BEL1, BLH9 and with KNAT1/BP [14]. Plants overexpressing *KNATM* displayed leaf serration phenotypes that resembled *saw1-saw2* mutants. Further functional studies revealed an antagonistic relationship between KNATM and SAWs [14]. 

A further role for KNOX in leaf lamina margin morphology was assessed by studying several *Arabidopsis* species, such as *A. arenas*, *A. halleri*, *A. suecica* and *A. lyrata*, which display a wide range of leaf lobing and serrations [69]. Different degrees of marginal outgrowth were associated with the expression of *STM-like* genes in leaves. It was proposed that the evolution of the unlobed shape of *A. thaliana* leaf may have involved *STM* loss of expression in the leaves and that cis-regulatory divergence contributed to margin diversification [69]. However, recent works reported on the occasional and feeble *STM* expression at the sinus of *A. thaliana* Columbia wild type leaves, suggest that STM may also be involved in serration formation in *A. thaliana* [70].

Several other genetic moieties could participate to KNOX-mediated leaf shape diversity as *KNOX1* repression in leaves is under epigenetic regulation at the chromatin level [21]. Indeed, multiprotein complexes are involved such as: (a) the chromatin remodeling factor Histone Cell Cycle Regulation defective homolog A (HIRA) [71] that includes AS1, a myb transcription factor, and AS2, a LATERAL ORGAN BOUNDARY DOMAIN (LBD) protein; (b) the POLYCOMB REPRESSIVE COMPLEXES (PRCs) 1 and 2 [72]; (c) the LEUNIG-YABBY complex [73]; (d) the transcriptional repression complex that contains the co-repressor TOPLESS (TPL) and TPL-related (TPR) that interacts with AS1 [74]. Interestingly, several TALE homeobox proteins contain the Ethylene-responsive element binding-factor-associated amphiphilic repression (EAR) motif [75], a consensus sequence that identifies transcription factors acting as repressors of gene expression and mediates physical interactions with TOPLESS co-repressors [76]. Therefore, interactions of TALEs with TPL and TPR co-repressor complexes could change their transcription factor properties from gene expression activators to repressors.

Additional transcription factors, other than TALE homeodomain, play a pivotal role in leaf margin morphology in *A. thaliana*. Investigation on the evolution and specific roles of *NAM/CUC* (*NO APICAL MERISTEM* and *CUP-SHAPED COTYLEDON*) meristem boundary genes in leaves showed that *CUC2* is essential for margins morphology of Arabidopsis wild type plants [77]. Inactivation of *CUC3* can suppress serrations, indicating a role for this gene in leaf shaping. Moreover, the lobed leaf phenotype of plants that overexpress *KNOX1* genes was suppressed in *cuc2-3* mutants, suggesting that CUCs act downstream of KNOX-induced leaf morphology alteration [77].

Further studies, which combined developmental genetics and computational modeling, showed that serration development is the morphological read-out of a spatially distributed regulatory mechanism, which creates reiterated activity peaks of auxin and the CUC2 transcription factor [70,78]. Therefore, CUC2 activity and auxin transport and signaling are regulated and integrated to sculpt leaf margin serrations in plant species. The contribution of *KNOX1* genes in this process is not completely clear in *A. thaliana* because of conflicting results on *STM-like* gene expression in wild type leaves [70,78].

### 3.3. TALE Genes in the Control of Compound Leaf Morphology

**Figure 3:**
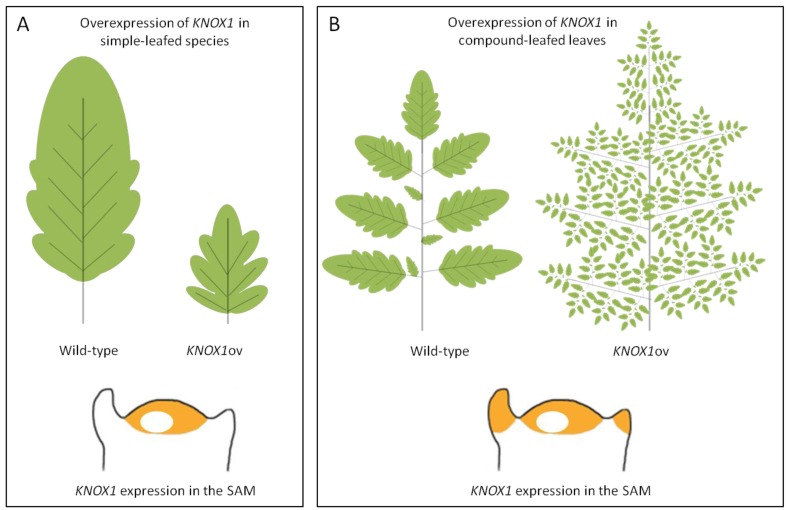
Overexpression of *KNOX1* genes in plants with simple or compound leaves. Schematic representation of the lobed leaf phenotype of Arabidopsis plants that overexpress *KNOX1* genes (**A**) or the supercompound leaf phenotype due to *KNOX1* overexpression in tomato (**B**). In the lower part of the panels the expression of *KNOX1* genes in the shoot apical meristem (SAM) of plants with either simple (**A**) or compound (**B**) leaves is schematized. *KNOX1* expression domains are shown in orange.

Tomato (*Solanum lycopersicum*) [26,29] and the close Arabidopsis relative *Cardamine hirsuta* [24] have been extensively studied to unravel genetic and molecular mechanisms at the base of leaf dissection. The first evidence of a role for TALEs in compound leaf development derived from overexpression studies of the maize *KN1* in tomato. In these plants, two to four additional reiterated rounds of ramification of the blade were induced, generating supercompound leaves bearing thousands of leaflets [79] (Figure 3B). Most compound-leafed species, differently from species with simple leaves, recapitulate *KNOX1* gene expression in leaf primordia after the initial downregulation at P0 (Figure 3) [22,27]. Expression of *KNOXs* during leaf primordia development has been correlated to the maintenance of an indeterminate state that would prompt the leaf to undertake morphological processes for leaflet production [55]. 

However, a set of studies on tomato, including the *KN1* overexpression in mutants with different levels of leaf dissection [79] and the switch of KNOX1 proteins into repressors combined with the time-regulated expression in leaf primordia [55], revealed that: (a) the sensitivity to KNOX1 action varies during leaf development; (b) the KNOX1 activity is restricted to the developmental window of primary morphogensis [38]. The extent of this organogenesis window is defined by the antagonistic activities of maturation promoting (e.g., TCP transcription factors, gibberellins, florigen) and maturation delaying factors (e.g., KNOX1, cytokinins) [25,26,29]. Additional evidences for a TALE role in compound leaf development arose from the molecular characterization of a tomato single semidominant locus, named *Petroselinum* (*Pts*), and the tomato classical mutant *bipinnata* (*bip*). Both the *Pts* locus and *bip* mutations conferred similar phenotypes which consisted of increased primary and secondary leaflet production, as well as development of tertiary and quaternary leaflets, not observed in wild type tomato [80]. *Pts* resulted to be a gain-of-function mutant of a novel *KNOX* lacking the homeobox, homologous to the Arabidopsis *KNATM*, whereas *bip* was a loss-of-function mutant of a *BLH* gene. PTS was demonstrated to antagonize KNOX1/BIP function, thus showing that alterations in BLH/KNOX heterodimers homeostasis can affect compound leaf development. 

In the absence of *KNOX* loss-of-function tomato mutants, the final confirmation that endogenous KNOX regulated leaf lamina dissection in compound-leafed species was provided by studies in *Cardamine hirsuta.* The partial inactivation of the *STM* gene converted the compound leaf into a simple leaf, thus demonstrating that KNOX proteins are necessary for compound leaf development [81]. 

Besides *KNOX* homeobox genes, the *NAM/CUC* transcription factors also participate in compound leaf development. Refined experiments of gene silencing and mutant analyses were conducted in four distantly related eudicot compound-leafed species. Reducing the function of *NAM/CUC* boundary genes led to the suppression of blade margin outgrowths and the production of few and fused leaflets [25]. It was therefore proposed that *NAM/CUC* genes promote formation of a boundary domain that delimits leaflets [25].

### 3.4. ILRC Subgroup of Legumes: An Exception to the TALE Rule?

As previously stated, *KNOX1* expression is downregulated at the site of leaf primordia initiation in both simple- and compound-leafed species. However in most plants with compound leaves, *KNOX1* expression is re-activated at the leaf primordium and plays a role in leaflet formation. The pattern of *KNOX1* gene expression in several vascular plants revealed a correlation between *KNOX* expression and leaf complexity [22]. However, a specific Inverted Repeat Lacking Clade (IRLC) of legumes, which comprises pea and *Medicago* genus, was suggested to follow alternative pathways that did not involve reactivation of *KNOX1* genes in leaves. Pea *KNOX1* are excluded from compound leaves and KNOX1 proteins were not detected using KNOX1 antibodies in leaves of the IRLC legume group [82]. However, three *KNOX1* mRNAs were detected in trifoliate developing leaves of *Medicago truncatula* [18]. 

**Figure 4:**
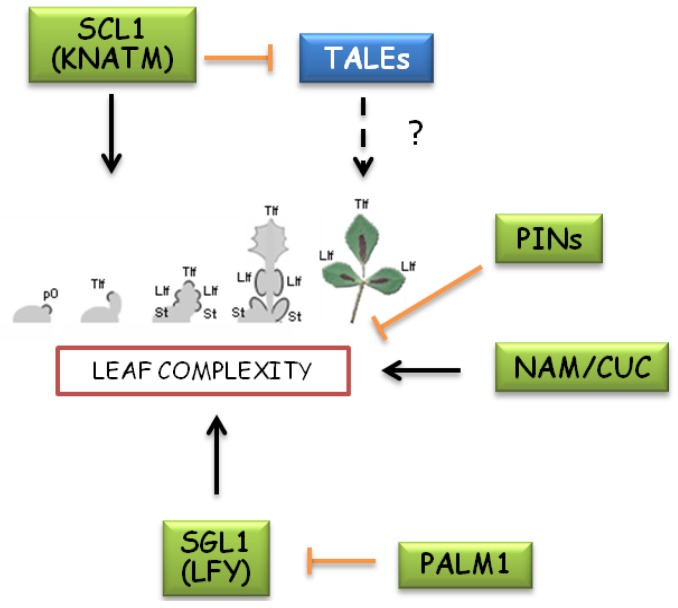
Genetic pathways involved in compound leaf development of *Medicago truncatula*. Leaf development in IRLC clade of legumes, including *Medicago* genus, seems to involve pathways alternative to KNOXs. *M. truncatula* mutations affecting leaflet number identified homologs of Arabidopsis *LFY*, *CUC*s and *KNATM* as genes promoting leaf complexity. A novel Cys(2)His(2) zinc finger transcription factor (*PALM1*) acts as a repressor of *SGL1/LFY* to reduce leaf complexity. Differently from *Cardamine hirsuta*, mutations in *PIN-like* genes result in increased formation of leaflets. The role of TALEs in *Medicago* leaf morphology remains unclear due to conflicting data regarding detection of mRNA in developing leaves and the effect of *KNOX1* overexpression on leaf complexity.

Despite conflicting results on *KNOX1* expression in *M. truncatula* leaf primordia, genetic studies implicated alternative pathways for the control of compound leaf morphology in these species (Figure 4). In pea, mutants of the Arabidopsis *LEAFY* (*LFY*) ortholog *UNIFOLIATA* (*UNI*) lacked tendrils and displayed simplified leaf complexity, besides floral defects similar to those of the Arabidopsis *lfy* mutant [83]. In *M. truncatula*, mutants in the *LFY* ortholog *SINGLE LEAFLET1* (*SGL1*) only developed simple leaves due to failure in initiating lateral leaflet primordia [84]. *LFY* is a plant-specific transcription factor that promotes the floral fate of meristems and is found as a single gene in most land plant species. Downregulation of *LFY* homologs in species outside of IRLC was also shown to induce a simplification of compound leaves [82]. This phenomenon might rely on a “cryptic” function of LFY to promote an indeterminate and meristematic state [85]. SGL1 function in *M. truncatula* leaf dissection was confirmed by the isolation of a negative regulator of *SGL1*, a Cys(2)His(2) zinc finger (*PALM1*) transcription factor. In loss-of-function *palm1* mutants, the lack of *SGL1* negative regulation resulted in an increase in the morphogenetic activity, which led to enhanced leaflet production [86]. Very interestingly, an additional *M. truncatula* mutant that displayed leaflet fusion and simplified leaf morphology resulted from loss-of-function of *FUSED COMPOUND LEAF1* (*FCL1*), an ortholog of the Arabidopsis *KNATM* [87]. As previously described, KNATM interferes with BLH/KNOX complexes homeostasis to promote KNOX1 morphogenetic activity by sequestering the SAW1,2 KNOX1 negative regulators. This raises the question whether KNOX1 can be somehow involved in leaf complexity in *M. truncatula*. Indeed, genetic analyses indicate that both *SGL1* and *FCL1* are required for the development of the higher complexity leaf displayed by *palm* mutant, suggesting the direct involvement of KNOX1 regulators in IRLC legume leaf development. However, loss-of-function mutants of *STM-like KNOX1* genes of *M. truncatula* did not display any simplification of the trifoliate leaf [88]. Overexpression of either *STM* or *MtKNOX STM-like* genes in *M. truncatula* altered shoot meristem activity and leaflet shape but failed to produce leaves with increased complexity [89]. However, overexpression of a tomato *KNOX1* gene in *M. sativa* was capable of producing extra leaflets on the rachis of the leaves [82].

The pattern of vasculature growth and leaf morphology varies during phase change from juvenile to adult with the reproductive phase, this phenomenon is termed heteroblasty. Genes involved in phase change and response to flowering, such as *L*FY and *FLOWERING LOCUS T* (*FT*) [90], or *SQUAMOSA PROMOTER-BINDING PROTEIN-LIKE* (*SPL*) transcription factors [91], genetically interact with *KNOX1* and *BLH*. This would suggest that TALEs may coordinate morphogenetic processes in response to environmental and developmental issues in different phases of shoot transition, through direct or indirect interactions with maturation promoting and maturation delaying factors. In this context, additional compound leaf mutants of *M. truncatula*, affected in homologs of genes involved in phase change, may help to further elucidate the cross-talk between TALEs and flowering transition factors.

## 4. TALEs Work at the Boundaries: How to Establish the Confines between Undifferentiated Cells and Organ Primordia

Leaves originally derived from modified branches so as to provide increased surface areas for light harvesting and photosynthesis. Leaf lamina growth follows the development of primary and secondary vascular strands. Simple and compound leaves mainly differ in degree and time at which lamina are initiated along the vascular growth axis, the latter may reflect the degree of determinate/indeterminate growth of the vascular tissues. In simple leaves, blade differentiation occurs early after the establishment of the primary vein, whereas in compound leaves the main vein branches several times before lamina differentiation occurs. Characterization of the *phantastica* (*phan*) mutant of snapdragon [92] and the *phabulosa* (*phb*) mutant of *A. thaliana* [93] showed that the establishment of dorsoventrality in the leaf primordium is an essential step for the initiation of marginal meristem activity leading to leaf blade formation. In *BLADE-ON-PETIOLE* (*bop*) mutants, the formation of ectopic outgrowths of blade tissue along the leaf petiole is accompanied by ectopic *KNOX* gene expression [94,95]. KNOX1 may therefore be involved in setting the boundaries between vascular identity or leaf blade identity. 

**Figure 5:**
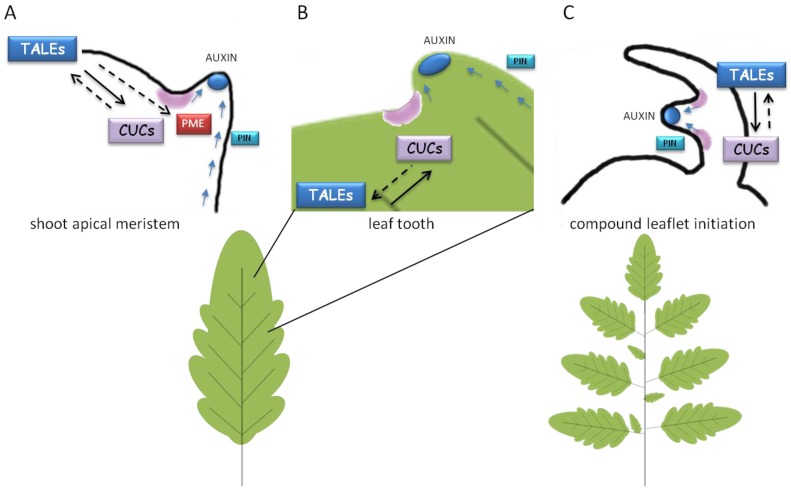
Common genetic pathways involved in lateral organ formation at the SAM (**A**), formation of leaf serrations (**B**) or leaflets in compound leaf development (**C**). PIN1-dependent auxin transport (blue arrows) leads to formation of auxin maxima (blue smarties) for organ/tissue outgrowth. Localized *CUC*s expression (in purple) and a positive feedback loop between CUCs and TALEs are required for setting boundaries and proper organ separation. TALE would maintain the indeterminate environment for morphogenesis whereas auxin and CUCs would ensure organ/tissue patterning and separation. In the SAM, TALEs would repress *PME* expression to prevent cell expansion. Continuous or discontinuous black arrows indicate either direct or indirect induction, respectively.

Establishing boundaries between undifferentiated cells and the sites of organ or tissue outgrowth constitutes a constant prerequisite in morphogenetic processes where differential areas of growth and polarity axis have to be established. Leaflet formation in compound leaves, and serrations in simple leaves, somehow recapitulate lateral organ primordia initiation occurring at the SAM. Common molecular players and regulators are involved in these processes (Figure 5). Recent evidence involved the so-called “morphogen” auxin, KNOX and CUCs as key interacting players in these morphogenetic events. The establishment of PIN-directed auxin gradients to form auxin maxima at the sites of organ or tissue outgrowth was shown to underlie organogenesis processes at the shoot apex [96], formation of compound leaf in *Cardamine hirsuta* [97] and tomato [98] as well as serration onset at the leaf margins [70,78].

The current model for leaf margin serrations predicts that the presence of CUC2 enables the reorientation of PINs whereas auxin, in turn, would inhibit CUC2 to stabilize the position of auxin maxima (Figure 5B). Therefore, the protrusion and indentations of the serrations would constitute a morphological readout of the sites of high auxin and CUC2 concentrations, respectively [78]. 

During compound leaf development, KNOX1 would contribute to maintain a prolonged undifferentiated cell fate in leaf primordia that would allow cells to respond to PIN-dependent auxin/CUC regulatory network for the reiteration of sites with no expansion (boundaries) and tissue outgrowth in correspondence to auxin peaks (Figure 5C). Surprisingly, different molecular mechanisms seem to occur in the marginal blastozone of the IRLC legume *M. truncatula* for compound leaf development (Figure 4). Mutations in *PIN1-like* genes, which displayed altered auxin maxima formation, resulted in increased formation of leaflets [99,100], thus showing an opposite effect with respect to other compound-leafed species where loss of auxin transport induced leaf simplification [71]. However, acropetal or basipetal growth of petioles, rachis and various orders of vein vasculature are species-specific and may take account of the different involvement of PIN-mediated auxin distribution in morphogenesis processes. 

Detailed studies on vascular axis growth in various compound-leafed species are envisaged in order to correlate gene expression pathways and function and to build predictive models that integrate morphological and molecular data. Identification of novel mutations in *TALE* and *PIN* genes in various members of IRLC legumes will help to unravel how the compound leaf pathway is established in these species. 

## 5. A Matter of Targets: the Secret Agents of TALEs

**Figure 6:**
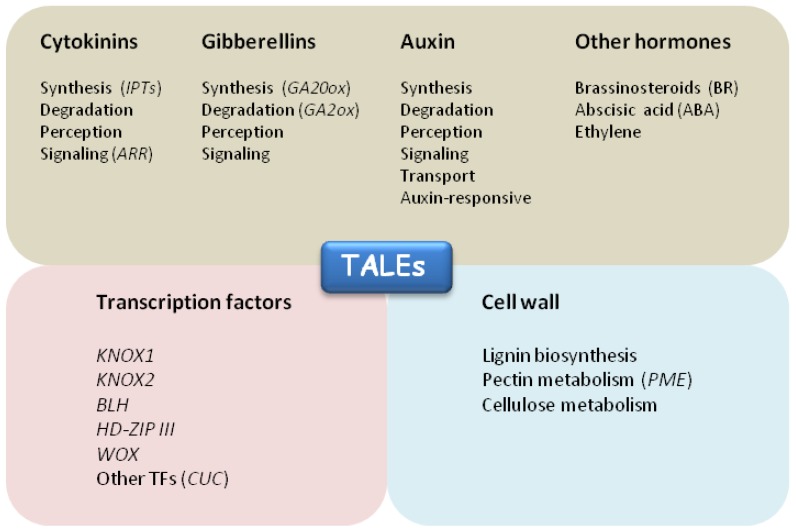
Functional categories of TALE target genes. Putative TALE direct targets belong to three main functional categories: hormonal pathways (grey box), transcriptional regulation (pink box) and cell wall metabolism (light blue box). KNOX1 positively regulate cytokinin biosynthesis and signaling through induction of *ISOPENTENYL TRANSFERASE (IPTs)* and *ARABIDOPSIS RESPONSE REGULATORS* (*ARRs*) genes. Low active gibberellins levels are maintained by KNOX1 through repression of GA biosynthetic genes (*GA20ox*) and induction of GA degradation by *GA2ox* genes. *CUC1* TF has been identified as a direct target of STM in Arabidopsis. Arabidopsis KNAT1/BP was shown to repress several genes involved in lignin biosinthesis. Arabidopsis BLH9/BLR negatively regulate *PECTIN METHYLESTERASE 5* (*PME5)* gene to repress cell expansion in the SAM. Recent studies of ChIP-seq and RNA-seq identified KN1 modulated and bound genes enriched in TFs and hormone metabolism, most notably auxin.

In order to understand TALE dependent cell behavior during development and diversification of morphogenetic structures, it is essential to identify many of their downstream genes. So far, only a few KNOX1 target genes have been characterized. Novel data from genome-wide studies are beginning to shed light on the complex regulatory network controlled by TALEs (Figure 6).

### 5.1. Links to Hormones

Cytokinins and gibberellins hormonal pathways were first identified as downstream targets of KNOX1 transcription factors [101,102,103,104,105,106]. Cytokinins (CK) are master regulators of plant development that stimulate cell division and induce shoot apical meristem formation in plant tissue culture. Several plant species overexpressing *KNOX1* genes accumulate high levels of cytokinins [101,102,107,108]. Independent studies have provided direct molecular evidence for the positive regulation of CK biosynthesis by KNOX1, through the induction of *ISOPENTENYL TRANSFERASE* genes, in Arabidopsis [104,105] and rice [109]. Lettuce plants overexpressing *KNAT1/BP* showed cytokinin overproduction and accumulation at the leaf margins, accompanied by ectopic leaves initiation [108] suggesting that KNOX1 would induce local changes in cytokinin content to increase the morphogenetic potential of leaf margins. This finding was confirmed by studies in tomato where ectopic expression of *IPT7* in leaves led to the formation of super-compound leaves whereas decreasing CK levels resulted in reduced leaf complexity [110]. 

Gibberellins (GA) promote polar cell expansion and organ outgrowth. The first evidence for a direct involvement of KNOX1 in regulating GA metabolism came from induction of the *STM* ortholog *NTH15* in tobacco. *NTH15* induction resulted in rapid repression of GA biosynthesis and transcript levels of the *GA 20-oxidase gene*, *NTC12* [76]. Recombinant NTH15 protein was also shown to bind specifically to a sequence in the first intron of *NTC12* [103]. Similarly, induction of the maize KN1 activity in Arabidopsis resulted in rapid repression of the *AtGA20ox1* transcript [111]. In *Solanum tuberosum*, the cooperative interaction between the TALE proteins StBEL5 and POTH1 directly repressed the potato *ga20ox1* gene by binding a specific promoter sequence [112]. The expression of *AtGA2ox2*, which encodes an enzyme involved in inactivation of bioactive GA was upregulated in response to STM in Arabidopsis and accumulated in leaves of the dominant maize mutant *Kn1-N* [113]. KN1 directly binds to a cis-regulatory element present in an intron of *ga2ox1* [106]. Thus TALE TFs maintain low quantities of active GA through direct repression of GA biosynthesis and up-regulation of GA catabolism. A similar antagonistic relationship exists between the *Tomato Knotted2* (*TKN2)* gene and *LeGA20ox1* in the dissected leaves of tomato. The dominant mutants *Mouse ears* (*Me*) and *Curl* (*Cu*), that misexpress *TKN2*, displayed increased leaf complexity and reduced *LeGA20ox1* transcript [111]. Constitutive GA signalling by the *procera* mutation suppresses the leaf dissection level in tomato and the increased leaf dissection seen in the *Me* mutant [111]. *PROCERA* encodes a DELLA growth repressor that is required at the early stages of leaf development both to promote leaflet formation and to restrict growth of the leaf primordia [114]. Thus the KNOX/GA interaction seems to be a component of a developmental module controlling indeterminacy in both SAM and leaves. 

Novel potential TALE target genes have been recently identified using ChIP-seq and RNA-seq high throughput technologies on maize *KN1* loss-of-function (*kn1-e1*) or gain-of-function (*Kn1-N*) mutants [115]. It was found that KN1 binds to several thousand loci that include 643 genes that are modulated in one or multiple tissues [115]. These genes were divided into functional categories and hormone metabolism was among those presenting the most significant enrichment. ChIP-seq results highlighted the preferential binding of KN1 near genes belonging to the GA, brassinosteroid (BR), and auxin pathways, but modulated genes were mainly enriched for the auxin pathway [115]. Cytokinin genes were not enriched as a group in this survey. However, few genes involved in CK perception and signalling, which included a homolog of the CK receptor *WOODEN LEG*, were modulated in *KN1* mutants. The latter suggested that KN1 may trigger CK synthesis and signalling by directly targeting only a few players in the CK pathway [115].

The identification of auxin as the main hormonal pathway regulated by KN1 was unexpected. Genes involved in auxin synthesis, transport, and signaling were both bound and modulated, particularly in leaves of *Kn1-N* [115]. The antagonistic relationship between KNOX1 and auxin has been suggested to operate in multiple contexts throughout plant development to promote organ initiation and the elaboration of organ boundaries [21,116]. Strikingly, lateral veins of *Kn1-N* displayed high auxin signaling, monitored by the auxin response sensor DR5, that overlapped with *KN1* accumulation [115]. This finding is a novelty since in the SAM high auxin activity occurs at sites of organ initiation where *KNOX1* genes are not expressed [117,118]. However, the identified target genes may be regulated in opposite ways by alternative KNOX/BLH complexes acting as transcriptional activators or repressors in different cells. It is worth noting that this survey identified several auxin response factors (*ARF)* and *Aux/IAA* genes as putative KN1 targets. Recently, a significant effort to unravel auxin signaling networking at the SAM was made using gene expression and interactome data, mathematical modeling and novel auxin markers [119]. This work provided evidence that the simple alteration of *ARF* and *Aux/IAA* global expression can establish a differential auxin sensitivity between the centre and the periphery of the SAM. It would be tempting to speculate that one of the as-of-yet unknown functions of TALEs could be the modulation of auxin sensitivity through the regulation of global *ARF* and *Aux/IAA* expression. KN1 was shown to bind *AUXIN SIGNALING F-BOX like* (*AFB-like*) genes that encode F-box auxin coreceptors in Arabidopsis [120]. The existence of such regulation may suggest a role of KN1 in auxin-dependent selective protein degradation through the ubiquitin proteasome system (UPS). Recently, we reviewed our current knowledge on the role of UPS in SAM function and integrated this survey with *in silico* analysis of available public databases to identify ubiquitin ligases (E3) that are expressed in specific areas within the SAM [121,122]. Interestingly, several substrate adaptors of SCF ubiquitin E3 ligases, including *AFB-like*, were predicted to express in specific domains of the SAM [97,98]. In addition, the distribution of other *AFB* co-receptor genes (such as *TIR1* and *AFB5*) was shown to vary between the centre and the periphery of the SAM [119]. Namely, the low *TIR1* expression at the centre and the high *AFB5* transcription in the internal tissues of the organ primordia suggest that these genes could also contribute to create domains with differential sensitivity to auxin [119]. 

In summary, KN1 can bind several genes involved in hormone biosynthesis, perception, degradation and signal transduction, as well as auxin transport, confirming and extending a pivotal role of TALEs in regulating hormonal homeostasis at multiple levels. However, activation or repression of target genes subsets may vary in different cells depending on the availability of BLH partners and other molecular interactions.

### 5.2. Transcription Factors

Transcript profiling by microarray analysis of transgenic lines in which *STM* was induced, identified *CUC1* as a direct target [123]. Hence, strict connection between *CUC* boundary genes and *KNOX1* was confirmed in morphogenetic processes where a mutual activation is necessary for the establishment of organ outgrowth. Many *NAM-ATAF1,2-CUC2 (NAC)* genes were also bound by KN1 and modulated in the ChIP-seq/RNA-seq on maize *KN1* loss-of-function or gain-of-function mutants [115], although this class of TF was not significantly enriched. Interestingly, KN1 direct targets were strongly enriched for transcription factors. The homeobox (HB) family prevailed among the bound TFs and comprised several *BLH* and 10 *KNOX* genes, including *KN1*. This points to the existence of complex regulatory feedback loops/networks controlling *KNOX* and *BLH* abundance and confirmed the importance of autofeedback regulation as previously demonstrated in rice for *OSH1* [124]. Other TF genes included members of the *HOMEODOMAIN LEUCINE ZIPPER* (*HD-ZIP*) and *MADS-box*. Among these classes, homologs of *REVOLUTA* (*REV*), *PHABULOSA* (*PHB*) and *YABBY* were also bound by KN1. The latter TFs are key regulators of leaf organ adaxial/abaxial polarity and blade outgrowth, and a regulatory link between these TFs and KNOX1 may account for coordination of lamina initiation and growth along the vascular growth axis in both simple and compound leaves. 

### 5.3. Cell Wall Proteins

Lignin biosynthesis was identified as a major target of the KNOX1 transcription factor KNAT1/BPin high density microarrays conducted on the *brevipedicellus* Arabidopsis mutant (*bp-9*) [125]. KNAT1/BP acts to repress lignin biosynthesis in order to prevent premature cell death as lignin deposition is a signature for irreversible cell differentiation [126]. Besides lignin biosynthetic enzymes, several other genes involved in the metabolism of cell wall polysaccharide components, such as cellulose and pectin, were altered in the *bp-9* mutant [125]. In transgenic lines where *STM* was induced [123], several cell wall genes were modulated besides *CUC1* [123], including genes involved in pectin, cellulose and lignin metabolism. 

This is very interesting in the light of recent findings that place pectin methylesterase genes (*PME*) downstream of the TALE BLH9/PNY transcription factor. A regulated demethylesterification of homogalacturonans (HGs), main constituents of the pectin, was shown to play a crucial role in cell wall loosening processes for organ primordia outgrowth at the shoot apex [61]. BLH9 would repress *PME5* gene expression in the inflorescence meristem to limit primordia formation. In meristem periphery, *BLH9* downregulation would allow *PME5* expression, thus triggering cell expansion events for primordia initiation [62]. 

These findings suggest that TALE homeobox proteins may modify cell wall properties by directly modulating genes involved in cell wall plasticity. In this respect, it would be interesting to study whether changes in pectin methylesterification status could be part of the mechanism involving auxin, KNOX and CUCs for leaflet formation in compound leaves and leaf margin morphogenesis. The characterization of the leaf phenotype of mutants in *BLH* and *PME* genes in compound leaf species would help in further dissecting the molecular mechanisms underlying leaf development and shape. 

## 6. Conclusions and Perspectives

Common genetic pathways have been unravelled in different developmental programs such as organ primordia initiation at the shoot apical meristem and the morphogenetic events that shape margins and complexity of leaves. TALEs play a key role in orchestrating the intricate regulatory network that underlies these processes. Recent genome wide analyses support and extend previous data pointing to TALEs involvement in controlling multiple hormonal signals and transcriptional regulators. Newly identified direct targets are enriched for transcription factors and genes participating in hormonal pathways, most significantly auxin. This is of particular interest given the key role of auxin in both initiation and elaboration of final morphology of leaves and vascular networks, the main processes affected in plants misexpressing TALE transcription factors.

Due to the conservation of the consensus DNA binding domain, thousands of loci are potentially bound by TALEs. A future challenge will be to identify what and how specific KNOX and BLH homo- and heterodimers regulate subsets of target genes in different cells as well as developmental context. To assess the role of different members of TALE family in leaf morphology and evolution will require the characterization of additional Arabidopsis mutants by fine phenotyping and gene expression profiling. Next Generation Sequencing (NGS) techniques will allow the extension of these analyses to non-model and crop species to increase our knowledge of the role of TALEs in leaf morphogenesis processes. Systems biology approaches combining gene expression data, interactome and mathematical modeling will be required to unravel the intricate network of genetic and molecular interactions that underlies TALE function.
